# Prosthetic Rehabilitation for a Patient With Purpura Fulminans Undergoing Quadruple Amputation: A Case Report

**DOI:** 10.7759/cureus.67384

**Published:** 2024-08-21

**Authors:** Yohei Tanaka, Shuichi Nakayama, Takaaki Ueno, Toshiki Miura

**Affiliations:** 1 Rehabilitation Medicine, JR Tokyo General Hospital, Tokyo, JPN; 2 Orthopedic Surgery, JR Tokyo General Hospital, Tokyo, JPN

**Keywords:** physical therapy, occupational therapy, lower limb prostheses, upper limb prostheses, purpura fulminans, prosthetic rehabilitation, quadruple amputation

## Abstract

Purpura fulminans (PF) is a rare and life-threatening syndrome characterized by cutaneous purpura resulting from disseminated intravascular coagulation (DIC) and intravascular thrombosis. PF typically develops as a severe complication of infections and is associated with high mortality rates. Effective treatment involves early recognition, aggressive resuscitation, appropriate antibiotic therapy, and the correction of coagulation abnormalities. Nevertheless, despite effective treatment, patients often ultimately require amputation of the affected limbs. This case report details the rehabilitation process of a patient with PF who underwent quadruple amputation.

The patient, a 48-year-old male, underwent quadruple amputation due to PF. After intensive care, he was admitted to a convalescent rehabilitation ward for prosthetic rehabilitation. The rehabilitation process combined physical and occupational therapy to facilitate independent living through the use of upper and lower limb prostheses and assistive devices. The patient presented with ulcerative lesions on the anterior surfaces of both knee joints upon admission. During treatment, he developed osteomyelitis of the right patella, which required intravenous antibiotics and limited rehabilitation to bed-based exercises. Following the administration of intravenous therapy, the prosthetist proceeded with the fabrication of lower limb prostheses. Subsequently, the patient was able to commence standing and gait training, and by the time of discharge, he was able to walk without a cane. Upper limb prostheses enabled independence in activities of daily living (ADLs) such as eating, dressing, and toileting. He was also able to perform cooking-related activities that are part of the instrumental activities of daily living (IADLs).

This case highlights the importance and achievable outcomes of rehabilitation for patients with PF who have undergone quadruple amputation. A multidisciplinary approach utilizing both upper and lower limb prostheses, as well as assistive devices, enabled significant functional recovery.

## Introduction

Purpura fulminans (PF) is a rare, life-threatening syndrome that presents with a characteristic pattern of cutaneous purpura resulting from disseminated intravascular coagulation (DIC) and intravascular thrombosis [1-5]. PF most commonly develops as a severe complication of infection, characterized by abnormal activation of procoagulant pathways, dysfunction of anticoagulant pathways, and endothelial damage [4-7]. Although meningococci are often described as the typical cause of infectious PF, other common organisms, including varicella, staphylococci, and streptococci, can also cause the condition [8-12]. This disease is particularly destructive, with mortality rates ranging from 50% to 90% [10,11]. The implementation of early recognition, aggressive resuscitation, appropriate antibiotic treatment, and the use of adjuvant therapies to correct coagulation abnormalities have significantly improved survival rates [12]. The necessity for fasciotomy is common, and surgical wound management, including debridement, skin grafting, and flap closure, is imperative in the majority of patients [13].

Occlusion of peripheral limb blood vessels often leads to eventual amputation [1,14]. A study involving 306 patients with PF reported that 51 out of 180 surviving patients (28.3%) required limb amputations, with a median of three limbs amputated per patient [15]. Another study reported that the rates of amputation involving all four limbs ranged from 25% to 33% in patients with PF [13,16].

While there are case series that describe the rehabilitation of patients who have undergone treatment for PF and subsequent limb amputations [12,17], the rehabilitation process has not been described in detail. Accordingly, we present a detailed account of the rehabilitation of a patient who underwent quadruple amputation resulting from PF. With the assistance of upper and lower limb prostheses and rehabilitation, this patient was able to resume an independent lifestyle. Our report includes photographic and video documentation of this case.

## Case presentation

Medical history

**Figure 1:**
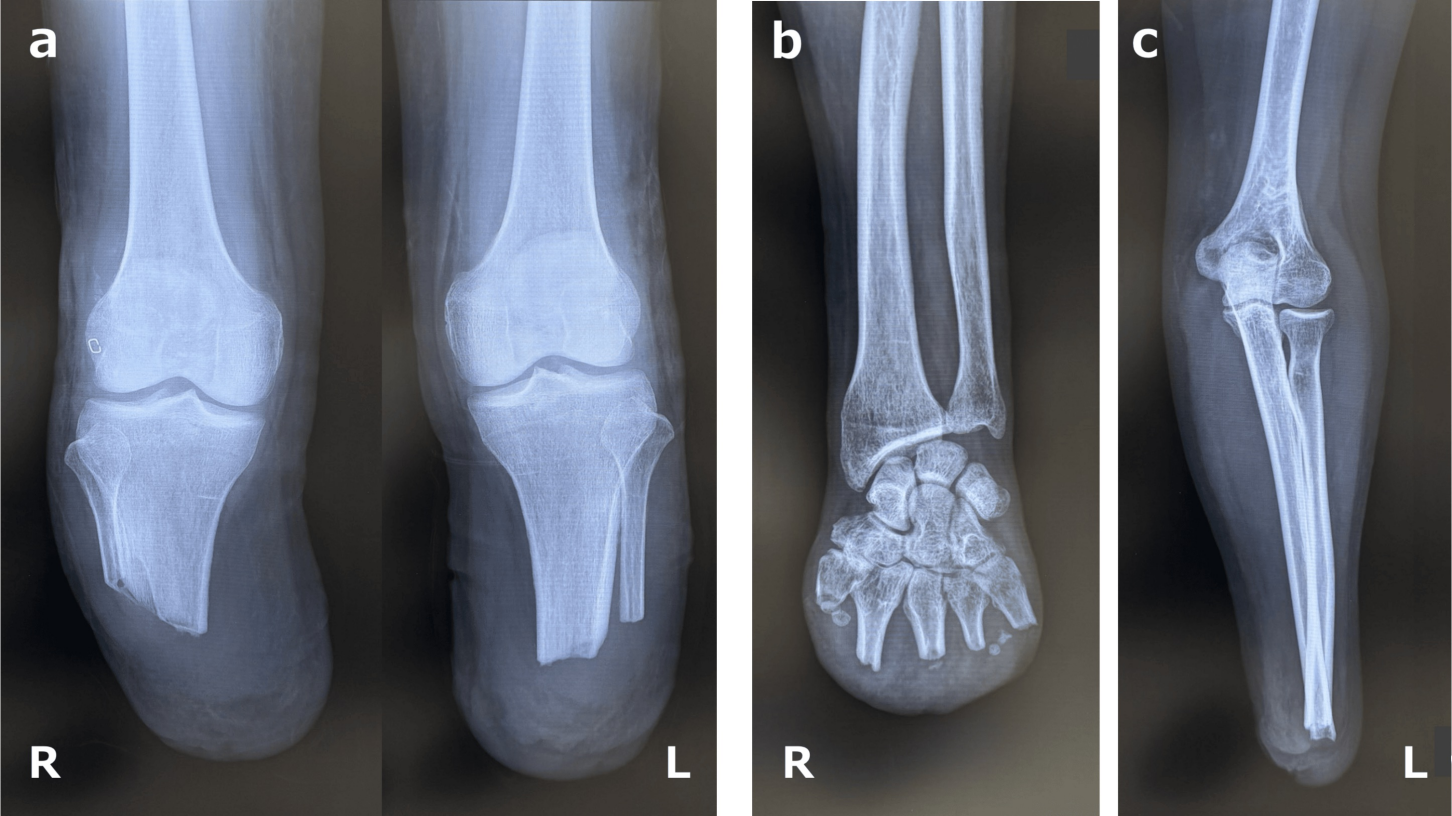
Plain radiographs of the stumps. (a) Bilateral below-knee amputation; (b) right partial hand amputation; (c) left below-elbow amputation.

The case presented is that of a 48-year-old male. He was in good overall health and had no significant medical history. Before his illness, he was a businessman living independently. He presented to the emergency and critical care center with acute deterioration, manifesting as diarrhea, vomiting, and decreased mobility. Upon admission to the hospital, he was diagnosed with septic shock and subsequently received intensive care. He exhibited purpura fulminans, with ischemic necrosis of all four extremities and the anterior surfaces of both knee joints. The anterior surfaces of both knees exhibited skin necrosis, which was treated with skin grafts. Eventually, one month after the onset of the disease, he underwent bilateral below-knee amputation, right partial hand amputation, and left below-elbow amputation due to peripheral gangrenous necrosis of the extremities (Figure 1). Three months after the initial diagnosis, he was admitted to our convalescent rehabilitation ward for prosthetic rehabilitation.

Physical examinations

**Figure 2:**
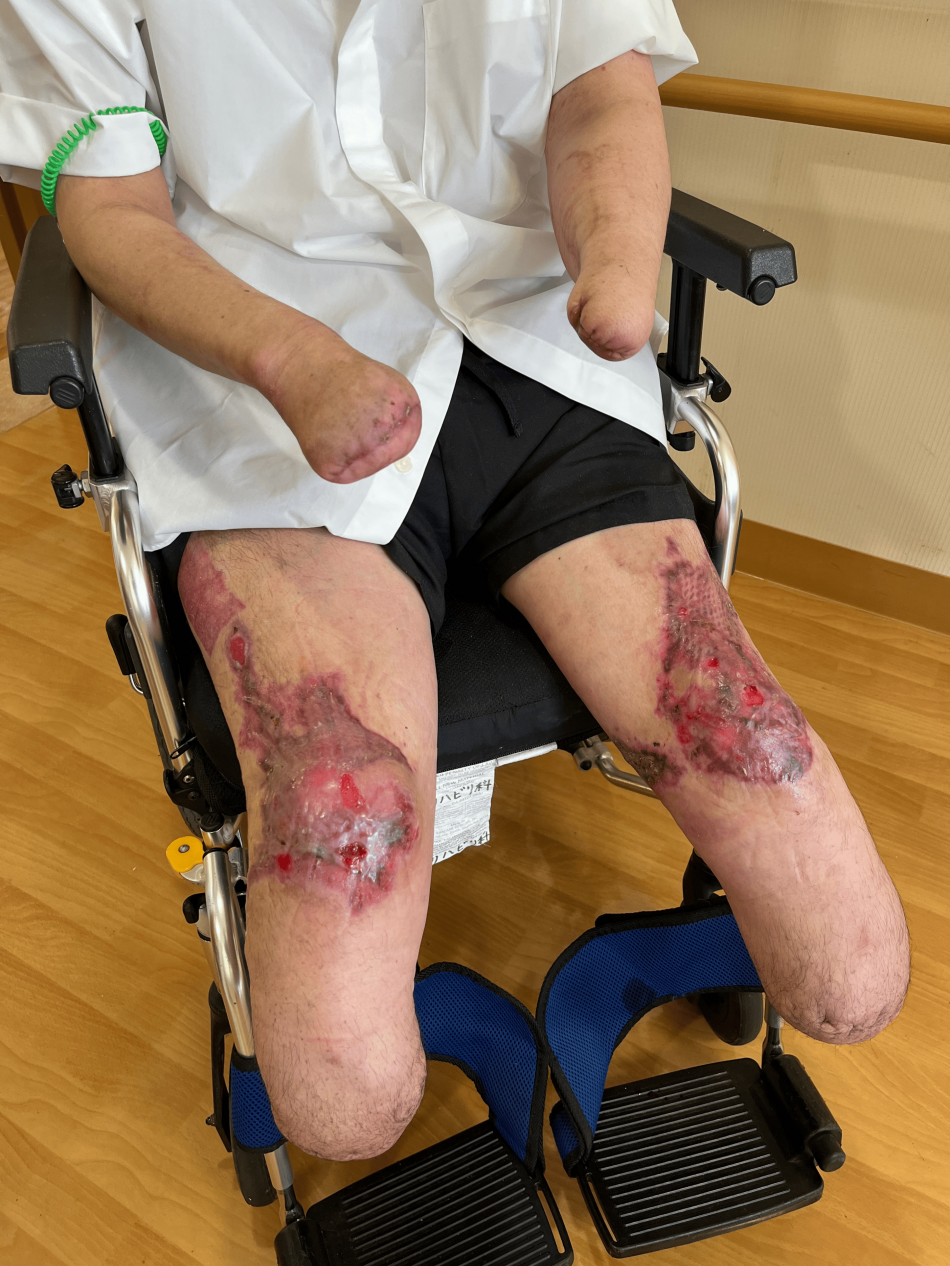
Skin lesions due to purpura fulminans on the anterior surfaces of both knees.

He was right-handed. Ulcerative lesions persisted on the anterior surfaces of both knee joints (Figure 2). The residual limb lengths of the upper extremities were 31 cm on the right and 23 cm on the left, measured from the lateral epicondyle of the humerus to the distal end. The residual limb lengths of the lower extremities were 17.5 cm on the right and 20 cm on the left, measured from the medial tibial plateau to the distal end.

The right elbow joint exhibited 120° flexion and -10° extension in active motion, and 130° flexion and 0° extension in passive motion. The right forearm demonstrated 60° pronation and 60° supination in active motion, with 75° pronation and 80° supination in passive motion. The right wrist showed 20° flexion and -10° extension in active motion, and 30° flexion and 0° extension in passive motion. The left elbow joint displayed 130° flexion and 0° extension in both active and passive motions. The left forearm exhibited 50° pronation and 20° supination in active motion, and 65° pronation and 30° supination in passive motion. The right knee joint had 80° flexion and -5° extension in both active and passive motions. The left knee joint presented 80° flexion and 0° extension in both active and passive motions.

Physical therapy

**Figure 3:**
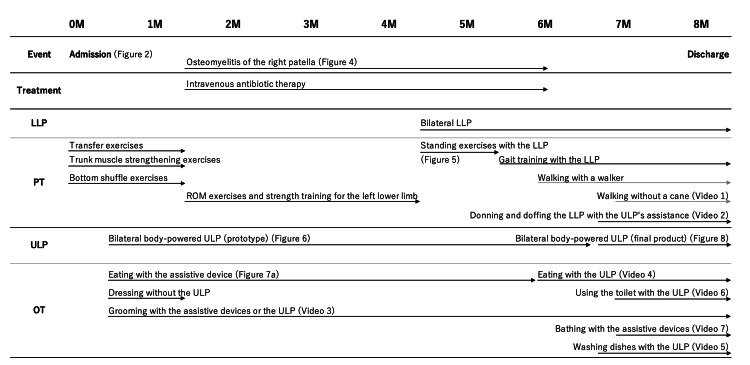
Progress in prosthetic rehabilitation. LLP, lower limb prostheses; PT, physical therapy; ULP, upper limb prostheses; OT, occupational therapy; ROM, range of motion; M, months.

The timeline and progression of physical therapy, occupational therapy, and prosthetic therapy are illustrated in Figure 3.

**Figure 4:**
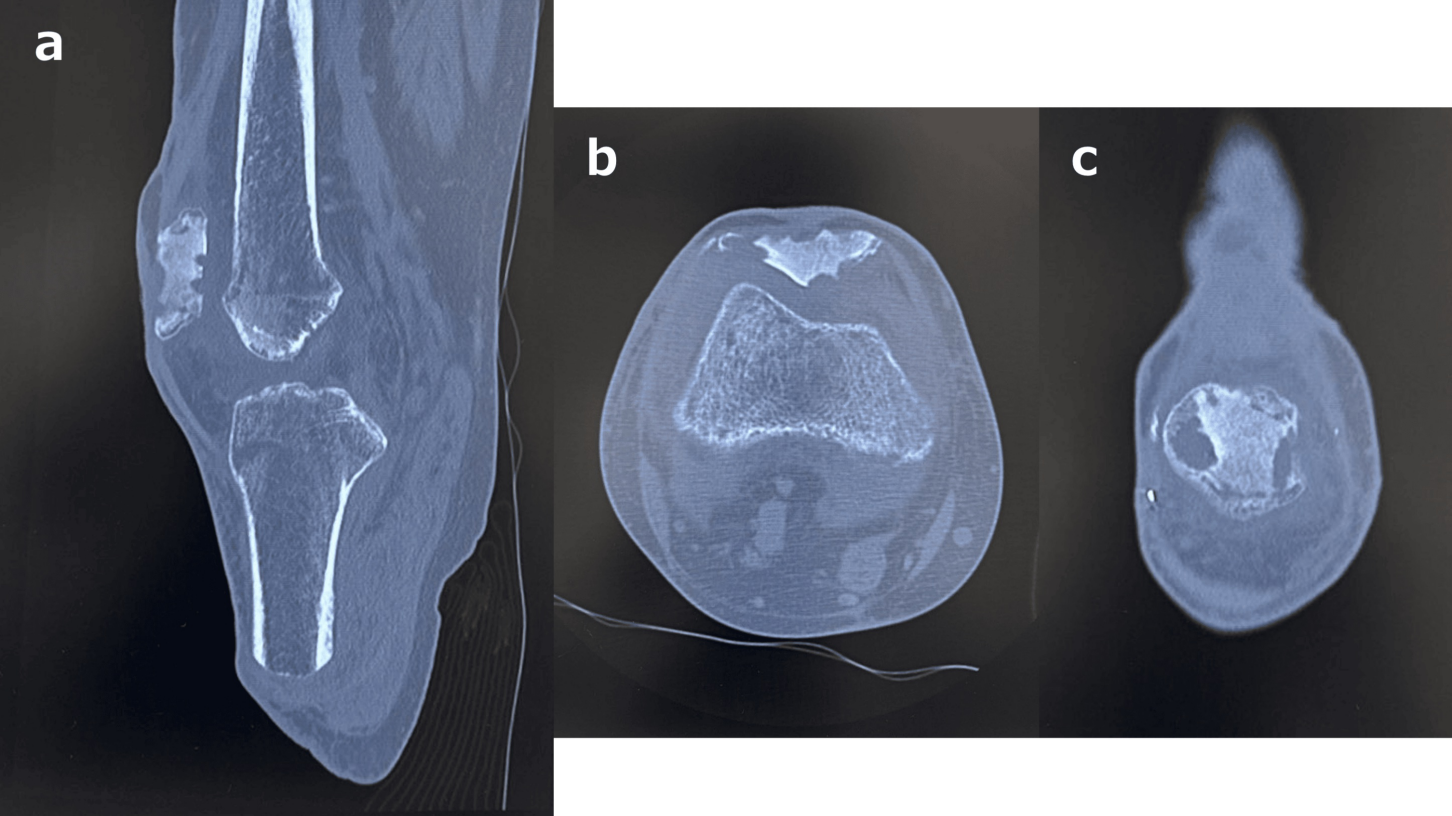
Computed tomography images of the osteomyelitis of the right patella. (a) Sagittal plane; (b) axial plane; (c) coronal plane.

Three months after the onset of the disease, the skin necrosis on the anterior surfaces of both knees had not yet completely healed. Following a 1.5-month stay in our hospital, the patient developed osteomyelitis of the right patella (Figure 4). The patient was treated with intravenous antibiotics for a total of 4.5 months. During this period, the patient was required to remain in bed, and rehabilitation was limited to joint range of motion exercises and strength training for the left lower limb. Intravenous antibiotic therapy resulted in the patient reaching a cured state; however, there was a delay in starting rehabilitation with lower limb prostheses.

**Figure 5:**
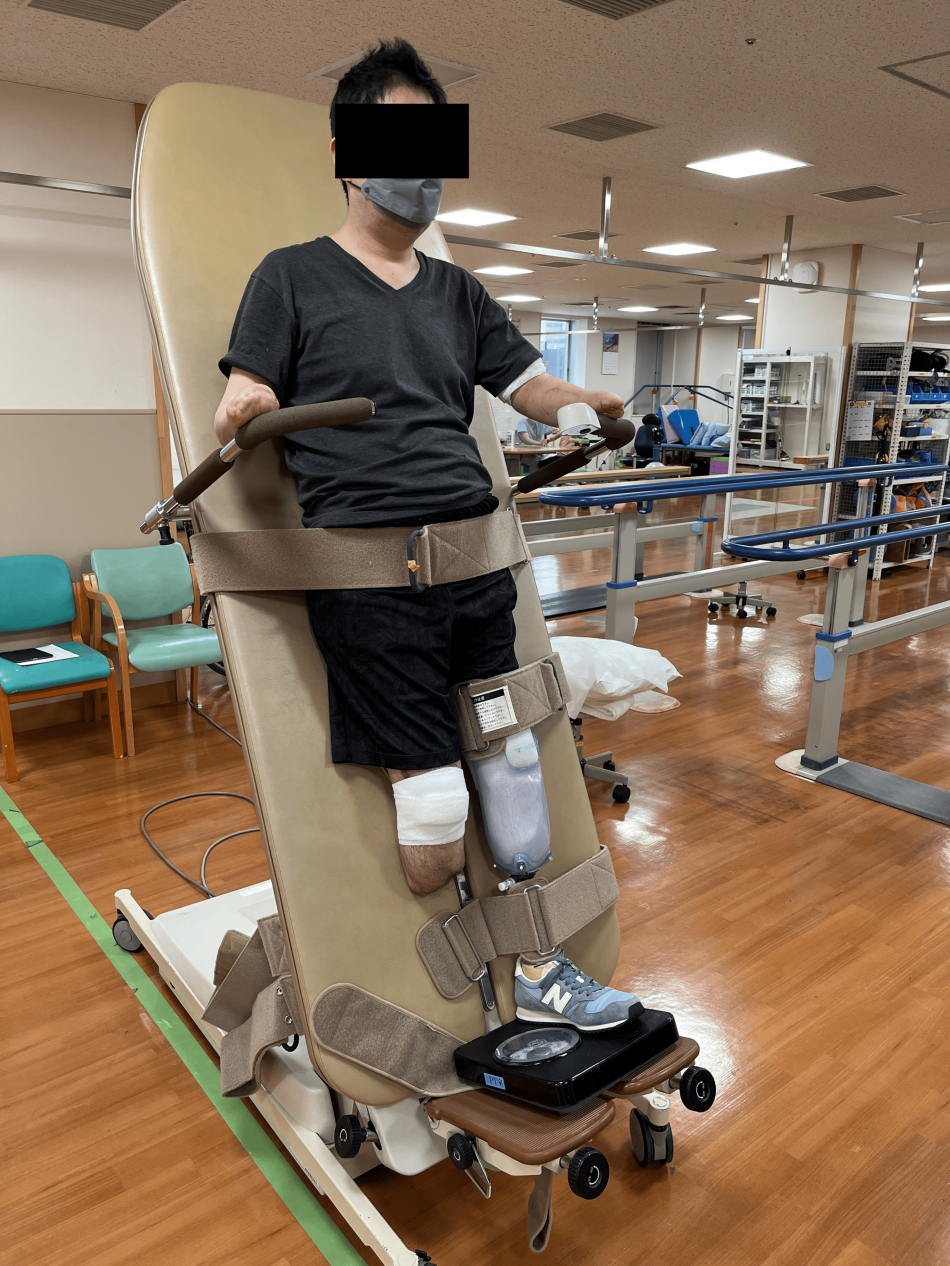
Standing training using a tilt table with a lower limb prosthesis fitted solely on the left side.

Rehabilitation with bilateral lower limb prostheses was initiated 4.5 months after admission. Due to the development of osteomyelitis in the right patella, the patient began standing training with the use of a tilt table (Figure 5). The patient's patellofemoral pain and inflammatory response were meticulously monitored, and gait training within the parallel bars was initiated. Subsequently, he began walking with the aid of a walker. By seven months post-admission, he was able to walk without a cane, albeit at a slower pace.

At the time of discharge, the range of motion of the right knee joint was 60 degrees of flexion and -5 degrees of extension, while that of the left knee joint was 90 degrees of flexion and 0 degrees of extension. His 10-meter walking speed was 0.80 m/s, and his six-minute walking distance was 240 m. A video of his gait is shown in Video 1.

**Video 1:** Walking with the bilateral lower limb prostheses.

The lower limb prostheses should be donned and doffed using the left body-powered upper limb prosthesis and the right bare hand. Using the handmade tube we prepared, he was able to independently don the silicone liner (Video 2).

**Video 2:** Donning the silicone liner and the lower limb prostheses.

Occupational therapy

**Figure 6:**
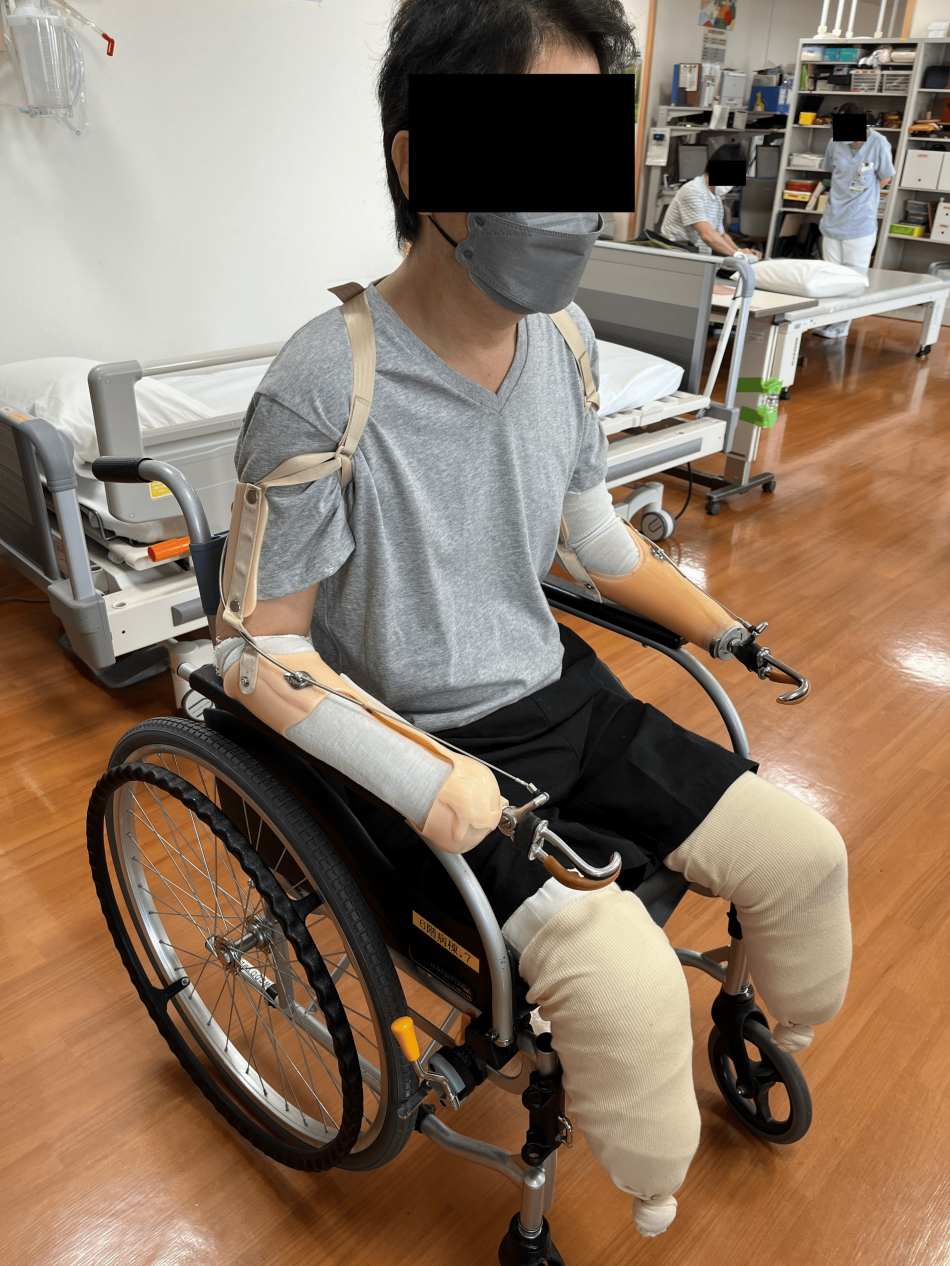
Bilateral body-powered upper limb prostheses (prototype).

Following admission, the prosthetist manufactured the initial bilateral body-powered prostheses for his upper limbs (Figure 6). However, in practice, the prototype partial-hand body-powered prosthesis for the right upper limb was found to be impractical due to its excessive length and lack of a flexion wrist.

**Figure 7:**
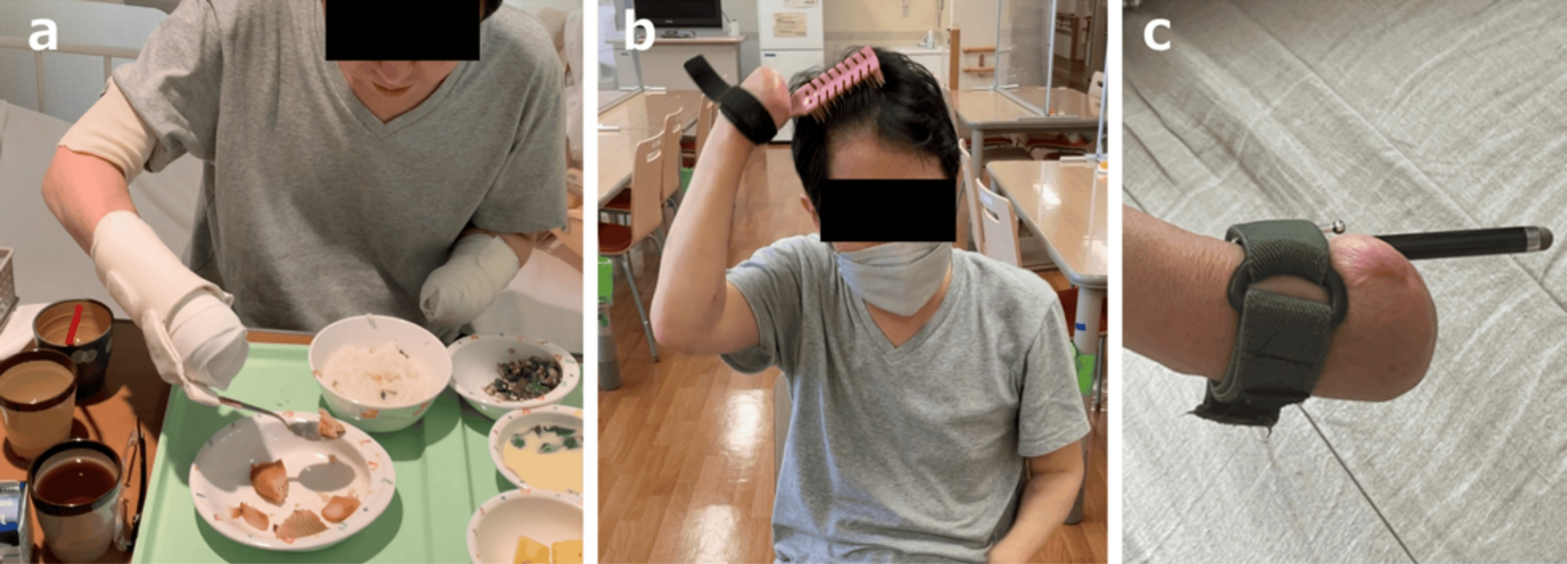
Assistive devices. (a) Eating with a spoon affixed to the assistive device; (b) hairdressing with a hairbrush affixed to the universal cuff; (c) using a stylus affixed to the universal cuff.

In the initial stage of the intervention, he was provided with an assistive device (Figure 7a) fabricated by his occupational therapist, which enabled him to eat meals using a spoon and fork. A universal cuff was wrapped around the end, to which a stylus for operating a smartphone and a brush for hairdressing was affixed (Figure 7b, 7c). He selected clothes without buttons, such as T-shirts and elastic waist pants, which he could don and doff without prostheses. He was able to carry out shaving using his left below-elbow body-powered upper limb prosthesis (Video 3).

**Video 3:** Shaving with the below-elbow body-powered upper limb prosthesis.

**Figure 8:**
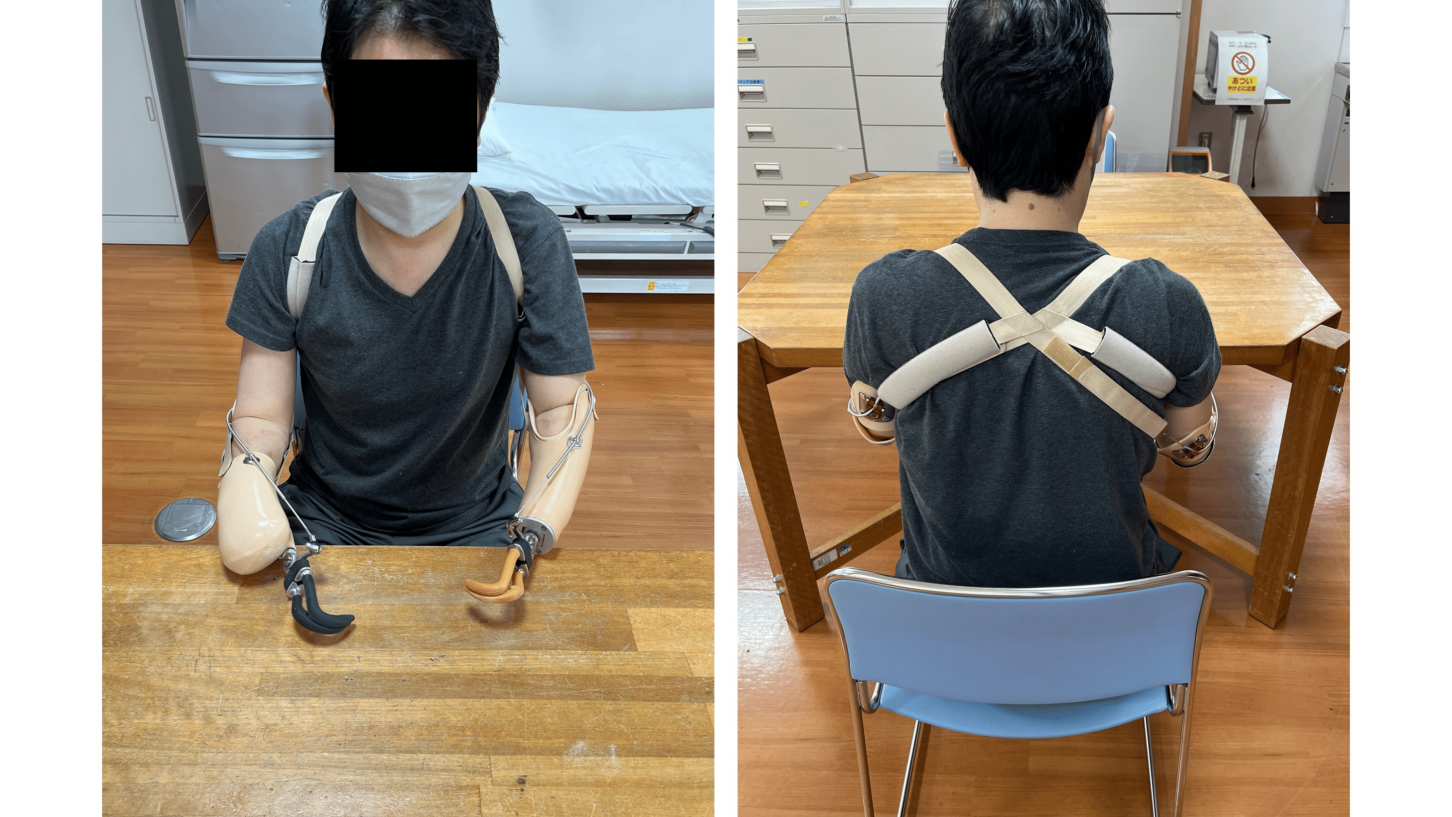
Bilateral body-powered upper limb prostheses (final product).

The body-powered upper limb prostheses were finally manufactured, as shown in Figure 8, after a process of trial and error. The left upper limb was typically fitted with a body-powered prosthesis, whereas the right upper limb was not always fitted with one and sometimes remained without a prosthesis. Consequently, the left and right upper limb prostheses were designed separately. The right body-powered upper limb prosthesis was redesigned with a shortened socket to maximize the range of motion for pronation and supination. The body-powered hook was relocated proximally due to the difficulty of using the longer prosthesis length. This enabled him to eat using his body-powered upper limb prosthesis (Video 4). Instrumental activities of daily living (IADLs), such as meal preparation and washing dishes (Video 5), could also be performed by using both the left and right-body-powered upper limb prostheses.

**Video 4:** Eating with the right body-powered upper limb prosthesis.

**Video 5:** Washing dishes with the bilateral body-powered upper limb prostheses.

He is now capable of performing toileting activities independently using the left body-powered upper limb prosthesis (Video 6). For activities associated with bathing, he is capable of performing the majority of requisite tasks using assistive devices (Video 7).

**Video 6:** Toileting activities with the left body-powered upper limb prosthesis.

**Video 7:** Bathing with assistive devices.

At the time of discharge, the patient exhibited the following ranges of motion in both active and passive motions in the upper extremities: right elbow flexion to 135 degrees and extension to 0 degrees, right forearm pronation to 90 degrees, and supination to 80 degrees, right wrist flexion to 55 degrees and extension to 60 degrees, left elbow flexion to 135 degrees and extension to 0 degrees, and left forearm pronation to 75 degrees and supination to 45 degrees.

## Discussion

The rehabilitation of patients who have undergone quadruple amputation due to purpura fulminans is a crucial aspect of their recovery process. The objective of rehabilitation is to enhance the patient's independence by facilitating the use of prostheses and other assistive devices [18]. Both physiotherapy and occupational therapy are necessary to improve mobility and establish activities of daily living with upper and lower limb prostheses and assistive devices.

Furthermore, many cases of purpura fulminans necessitate surgical intervention for the treatment of the affected skin area [13]. Similarly, in the present case, the treatment of this condition resulted in a prolonged period before rehabilitation could be completed. Specifically, a skin ulcer on the right knee developed into osteomyelitis of the patella, which contributed to the patient's inability to wear a prosthesis for an extended period and resulted in a prolonged hospital stay. Regarding the right knee joint, the patient's range of motion was diminished compared to that at the time of admission due to patellar osteomyelitis. Although range of motion exercises were administered, progress in the left knee joint was limited, possibly due to the delayed initiation of gait training, despite some improvement. Pain in the right knee joint impaired mobility, leading to the patient’s slow walking speed at the time of discharge.

This patient achieved independence in ADLs, including eating, dressing, and toileting, through the mastery of body-powered upper limb prostheses. Furthermore, he was able to don and doff his lower limb prostheses by himself. It is crucial for individuals with quadruple amputations to possess the ability to don and doff their prostheses independently, as this enables them to live a self-sufficient lifestyle. The use of a pin/lock suspension system with a silicone liner is beneficial due to its straightforward application and enhanced safety. Body-powered upper limb prostheses are instrumental in enhancing the independence of bilateral upper limb amputees. These prostheses are useful for maintaining feasible ADLs and IADLs, regardless of location.

In cases of bilateral upper limb amputation, both sides should be fitted with functional prostheses, including body-powered or myoelectric upper limb prostheses [12,17,18]. In cases of partial hand amputation, the use of assistive devices, such as a universal cuff, may also prove advantageous. The selection of the most appropriate prosthesis for the upper limb should be based on the length of the residual limb. In the present case, it was deemed appropriate to utilize a body-powered or myoelectric upper limb prosthesis on the left below-elbow amputation side and a body-powered upper limb prosthesis or an assistive device without a prosthesis on the right partial hand amputation side. Eventually, a body-powered prosthesis was chosen for the initial fitting of the left upper limb, given the assessment that insufficient time had elapsed since the amputation surgery and that the stump had not yet reached a sufficient level of maturation. In the future, with the objective of further expanding the patient's upper limb function, we are contemplating the introduction of a myoelectric upper limb prosthesis for the patient's left below-elbow amputation.

This case report is based on the Japanese healthcare system. Compared to other countries, Japan permits patients to be hospitalized for longer periods. Adequate rehabilitative care is provided during the course of hospitalization with the objective of facilitating patient independence.

## Conclusions

This case highlights the significant challenges and achievements in the rehabilitation of a patient with quadruple amputation resulting from purpura fulminans. The detailed rehabilitation process, involving both physical and occupational therapy, underscores the importance of a multidisciplinary approach to enhancing the patient’s independence and quality of life. Through the use of upper and lower limb prostheses and assistive devices, the patient was able to regain substantial functionality in activities of daily living, such as eating, dressing, and toileting. Despite the complexities associated with delayed rehabilitation due to complications such as osteomyelitis, the patient achieved a considerable degree of mobility and independence at discharge.

This case emphasizes the necessity for early and comprehensive rehabilitative care in similar patients and the potential benefits of incorporating advanced prosthetic technology such as myoelectric upper limb prostheses in future treatment plans. The prolonged hospital stay permitted by the Japanese healthcare system played a crucial role in providing adequate time for rehabilitation and achieving patient independence. Further research and case studies are recommended to optimize rehabilitation protocols and prosthetic use for patients with severe limb loss resulting from purpura fulminans.
